# Classes of Drugs that Mitigate Radiation Syndromes

**DOI:** 10.3389/fphar.2021.666776

**Published:** 2021-05-18

**Authors:** Ewa D. Micewicz, Robert D. Damoiseaux, Gang Deng, Adrian Gomez, Keisuke S. Iwamoto, Michael E. Jung, Christine Nguyen, Andrew J. Norris, Josephine A. Ratikan, Piotr Ruchala, James W. Sayre, Dörthe Schaue, Julian P. Whitelegge, William H. McBride

**Affiliations:** ^1^ Department of Radiation Oncology, University of California at Los Angeles, Los Angeles, CA, United States; ^2^ California NanoSystems Institute, University of California at Los Angeles, Los Angeles, CA, United States; ^3^ Department of Molecular and Medical Pharmacology, University of California at Los Angeles, Los Angeles, CA, United States; ^4^ Department of Bioengineering, Henry Samueli School of Engineering, University of California at Los Angeles, Los Angeles, CA, United States; ^5^ Department of Chemistry and Biochemistry, University of California at Los Angeles, Los Angeles, CA, United States; ^6^ Pasarow Mass Spectrometry Laboratory, University of California at Los Angeles, Los Angeles, CA, United States; ^7^ BCN Biosciences, LLC, Pasadena, CA, United States; ^8^ Department of Biostatistics and Radiology, Fielding School of Public Health, University of California at Los Angeles, Los Angeles, CA, United States

**Keywords:** radiation mitigators, hematopoietic acute radiation syndrome, gastro-intestinal acute radiation syndrome, delayed effects of radiation exposure, multi-organ disease syndrome, High throughput screening

## Abstract

We previously reported several vignettes on types and classes of drugs able to mitigate acute and, in at least one case, late radiation syndromes in mice. Most of these had emerged from high throughput screening (HTS) of bioactive and chemical drug libraries using ionizing radiation-induced lymphocytic apoptosis as a readout. Here we report the full analysis of the HTS screen of libraries with 85,000 small molecule chemicals that identified 220 “hits.” Most of these hits could be allocated by maximal common substructure analysis to one of 11 clusters each containing at least three active compounds. Further screening validated 23 compounds as being most active; 15 of these were cherry-picked based on drug availability and tested for their ability to mitigate acute hematopoietic radiation syndrome (H-ARS) in mice. Of these, five bore a 4-nitrophenylsulfonamide motif while 4 had a quinoline scaffold. All but two of the 15 significantly (*p* < 0.05) mitigated H-ARS in mice. We had previously reported that the lead 4-(nitrophenylsulfonyl)-4-phenylpiperazine compound (NPSP512), was active in mitigating multiple acute and late radiation syndromes in mice of more than one sex and strain. Unfortunately, the formulation of this drug had to be changed for regulatory reasons and we report here on the synthesis and testing of active analogs of NPSP512 (QS1 and 52A1) that have increased solubility in water and *in vivo* bioavailability while retaining mitigator activity against H-ARS (*p* < 0.0001) and other radiation syndromes. The lead quinoline 057 was also active in multiple murine models of radiation damage. Taken together, HTS of a total of 150,000 bioactive or chemical substances, combined with maximal common substructure analysis has resulted in the discovery of diverse groups of compounds that can mitigate H-ARS and at least some of which can mitigate multiple radiation syndromes when given starting 24 h after exposure. We discuss what is known about how these agents might work, and the importance of formulation and bioavailability.

## Introduction

Historically, tissue damage after exposure to low density ionizing radiation (IR) such as X-rays and γ-rays was ascribed to cell death resulting in large part from the generation of short-lived free radicals and their destructive effects on DNA, events that were widely considered to be determined within minutes to hours after exposure. The finding by the Walter Reed Army Drug Development Program in the 1950s that thiol-based free radical scavengers, from which WR2721 (Amifostine) emerged as the lead compound, non-selectively radioprotect normal tissues if given close to the time of radiation exposure, support this contention (Murray and McBride, 1996). Unfortunately, such radioprotectors are far less effective if given after exposure, which is an essential requirement for radiation mitigation in the context of radiological accidents or terrorist action. Government agencies have outlined a general framework for the development of such mitigators in animal models; agents must be efficacious when administered at least 1 d after IR exposure by a route suitable for mass administration. Despite these tight restrictions, promising mitigators have emerged that are active against potentially lethal, acute radiation syndromes (ARS) in animal models that closely mimic the human situation (Bond et al., 1965; Kim et al., 2009; Burdelya et al., 2012; Medhora et al., 2012; Himburg et al., 2014; Jiang et al., 2014; Cohen et al., 2016; Micewicz et al., 2017; Steinman et al., 2018), and in many ways they challenge historical radiobiological concepts that DNA damage and repair, and rapid apoptosis, is essentially complete within 24 h since mitigation of radiation-induced syndromes only start at this time point.

ARS were first characterized in radiobiological studies performed largely in inbred mice shortly after WWII. As whole-body irradiation (WBI) doses were increased, the dose causing 50% mortality (LD50) of mice plotted against mean/median survival time (MST) was found to be discontinuous with mortality being expressed within several dose-time windows, each with a characteristic pathogenesis (Quastler, 1945a; Quastler, 1945b; Quastler, 1945c; Austin et al., 1956; Sacher and Grahn, 1964; Bond et al., 1965). Lethality in the lowest dose range occurs between 10 and 30 d and can generally be ascribed to hematopoietic failure (H-ARS). An increase in dose precipitates an earlier phase of mortality at around 5–9 d due to gastrointestinal damage (GI-ARS). H-ARS and GI-ARS are primarily due to depletion of highly proliferative stem/progenitor compartments and failure of these tissues to maintain their functional compartment (Schaue and McBride, 2019; McBride and Schaue, 2020). The MST therefore reflects the tissue turnover time. A third, cerebrovascular/central nervous system syndrome (CVS/CNS-ARS) was identified 1–2 d after very high doses of WBI that is mechanistically different in being marked by brain edema, hemorrhage, and inflammation. Many variables may influence these morbidity and mortality patterns after WBI, including infection, immunosuppression, coincidental trauma, burns, and other exigent circumstances. In addition, individuals who survive ARS often develop delayed effects of acute radiation exposure (DEARE) that are largely associated with chronic inflammation and may display a multi-organ disease syndrome (MODS) with a shortened life span (Williams and McBride, 2011; Micewicz et al., 2019).

Although colony stimulating factors such as Neupogen (G-CSF), Neulasta (pegylated G-CSF), and Leukine (GM-CSF) have been approved by the FDA for H-ARS mitigation in humans (Singh and Seed, 2020), their efficacy may be limited with respect to other ARS and DEARE syndromes (Farese et al., 2019). They may even exacerbate radiobiological damage in some tissues (van Os et al., 2000; Li et al., 2015). Since WBI causes multi-organ damage, mitigators should be agnostic with respect to tissue type, which might require that they act through shared highly conserved pathways. Rebalancing all damaged tissue systems might prevent a later downward spiral into MODS. Additional advantageous properties might be selectivity for radiation tissue damage without other normal tissue effects, and formulation in effective doses that are easily administered and not highly dependent on time and route of administration.

We have used high throughput screening (HTS) and structure-activity relationship (SAR) analyses to identify classes of novel mitigators with at least some of these attributes when tested *in vivo* (Micewicz et al., 2017). Remarkably, the HTS platform employed simple radiation-induced apoptotic death of a CD4^+^CD8^+^ T lymphocyte line (Kim et al., 2009; Kim et al., 2011; Micewicz et al., 2017), suggesting that common core pathways are being targeted (Lant and Derry, 2014; Contreras et al., 2018).

We initially reported on results of a screen of 65,000 bioactive compounds that identified several classes of agents that could mitigate both radiation-induced lymphocyte apoptosis *in vitro* and H-ARS *in vivo*. These included purine nucleosides and multiple tetracyclines and fluoroquinolones, but not other antibiotics (Kim et al., 2009; Kim and McBride, 2010). For the antibiotics, mitigation was unrelated to anti-microbial activity and SAR analysis identified them all as belonging to a class possessing a common ring structure. We then interrogated small chemical libraries of 85,000 compounds using the same screening platform and reported on a class of novel 4-nitrophenylsulfonamide compounds (NPS), in particular piperazine derivatives (NPSP), with a lead compound NPSP512 that uniquely mitigated multiple ARS and late DEARE (Micewicz et al., 2017; Micewicz et al., 2019) including H-ARS, GI-ARS, radiation-induced pneumonitis and pulmonary fibrosis, neurological motor, sensory and memory deficits, and DEARE (Micewicz et al., 2017; Micewicz et al., 2019; Bhat et al., 2020; Duhachek-Muggy et al., 2020), as well as radioprotecting mice against H-ARS when given prior to WBI and displaying anti-tumor activity (Micewicz et al., 2017; Bhat et al., 2020; Duhachek-Muggy et al., 2020). Here, we describe other classes of mitigators from the HTS chemical screen and other sources. Over 20 H-ARS mitigators are described and their properties compared and discussed. We also describe the challenge in formulating the NPSP512 compound that led to analog synthesis designed to improve its pharmacological properties.

## Materials and Methods

### HTS Assay

The HTS assay has been described previously (Kim et al., 2009; Micewicz et al., 2017). In brief, TIL1 CD4^+^CD8^+^ lymphocytic cells of C3H murine lineage (McBride et al., 1992) were grown in MEM medium with 10% fetal calf serum and irradiated with 2 Gy. One hour later, 85,000 compounds were individually added from the ChemBridge DIVERSet (San Diego, CA, United States) or the Asinex (ASN) or Asinex Targeted (AST) libraries (Moscow, Russia). The AST library is configured around inhibitors of GPCRs (14 groups), kinases (six groups), ion channels (four ligand dependent groups), and proteases, while the others cover a broader pharmacophore space. Compounds were added at 10 µM final concentration in 1% DMSO. Mitigation of radiation-induced apoptosis was assessed at 24 h by measuring ATP release (ATPlite, Perkin-Elmer, MA, United States). Based on the 99% confidence limits compounds that increased viability to above 130% of the irradiated (diluent) controls (100%) were retested over a range of concentrations using both ATPlite and Annexin/P.I. (Fisher, Carlsbad, CA, United States) for apoptosis. For *in vivo* assays, compounds were obtained from ChemBridge (San Diego, CA, United States) or synthesized in house (MJ, PR). Purity and stability were assessed by LC/MS and NMR. All chemicals were stored in 15 µL DMSO or freshly suspended in 1 ml of 2 % Cremophor EL in water, and injected s. c. in the flank in 0.2 ml volumes, unless otherwise stated. All control mice received the same diluent as the experimental groups. Other vehicle formulations are noted in the text. The HTS data are archived in The Collaborative Drug Discovery Vault (https://app.collaborativedrug.com/vaults/425/searches/new) and freely available.

### Similarity and Substructure Analyses

Data were compared for similarity and chemical features on a Collaborative Drug Discovery vault platform (CDD™, Burlingame, CA, United States). The entire library was ranked by structural similarity to a referenced hit based upon the Tanimoto coefficient, excluding coefficients <0.7. Hits and non-hits within the library with similar structures were identified and maximal common substructure analysis performed to determine minimal core moieties (Chemaxon, Boston, MA, United States).

### *In Vivo* Testing

C3Hf/Sed//Kam and C57Bl/6 gnotobiotic male and female mice were bred at UCLA in our Radiation Oncology AAALAC-accredited facility. Where indicated, Foxp3^DTR/EGFP^ and Nrf2^−/−^, both on the C57Bl/6 background were used with the goal to monitor ARS under conditions of limited immune suppression and/or limited anti-oxidant defense, respectively. Foxp3^DTR/EGFP^ transgenic mice (a gift from Dr Chatila) have the Diphtheria toxin receptor under the control of the Foxp3 promoter (a master regulator of regulatory T cells, i.e., Tregs) that allows the conditional depletion of Tregs upon exposure to Diphtheria toxin (DT). In order to deplete Tregs mice were i.p., injected with DT at 1 µg/mouse (Sigma-Aldrich) every 3 days starting 2 days prior to WBI for a total of three injections while on maintenance antibiotics: Sulfatrim, 1 g/L Kanamycin and 1 g/L Ampicillin in drinking water (Haribhai et al., 2011). Nrf2^−/−^ came from the Jackson Labs (Bar Harbor, ME, United States). All mice were housed at four per cage and randomized to experimental groups of *n* = 8 at around 9–12 weeks of age, restricting the weight to match groups, e.g. males were 28–30 g when used. All IACUC-approved protocols and NIH guidelines were adhered to and defined criteria for premature humane euthanasia were strictly followed.

WBI was performed on unanesthetized and unrestrained mice using an AEC Gamma Cell 40 cesium irradiator (Cs-137) at a dose rate of around 60 cGy/min or a Gulmay Medical RS320 Irradiation System X-ray unit operated at 300 kV (Gulmay Medical Ltd., Surrey, UK) with a permanent 4 mm Be filter and 1.5 mm Cu and 3 mm Al giving a HVL of 3 mm Cu. Institutional probit analyses were used to determine the dose that would cause lethality of 70% of mice within 30 d (LD_70/30_). Dosimetry used a Capintec ionization chamber calibrated to NIST standards and film (GAFCHROMIC EBT2, International Specialty Products, Wayne, NJ, United States) to check that deviations in the field uniformity is <5%. Partial body irradiations used 300 kV X-rays (Gulmay, Surrey, UK) with anesthesia and Cerrobend (1 cm) to prevent exposure to unirradiated body parts. Mice were anaesthetized with an i.p. injection of 80 mg/kg Ketamine (Putney, NADA#200–073) and 4 mg/kg Xylazine (AnaSed, NADA# 139–236; Lloyd labs #4811).

We have outlined the radiation syndromes our mice experience in considerable detail (Micewicz et al., 2017; Bhat et al., 2020; Duhachek-Muggy et al., 2020). In brief, H-ARS mortality occurred during the “classic” time frame between days 10–30 after exposure. Hematological damage is confirmed by Hemavet CBC observations made on blood from a separate group of identically treated mice so as not to influence survival data. To rule out infection as a cause of death, plasma is cultured for aerobes and anaerobes, which is facilitated by the use of gnotobiotic mice, which have a limited flora that does not change over the experimental period. Active mitigators allowed for CBC recovery around day 10–14, especially for lymphocytes and neutrophils. GI-ARS occurs 5–9 days after abdominal exposure and is accompanied by epithelial denudation that is prevented by mitigators. Lethality after lung irradiation in C3H mice is due to pneumonitis and occurs 80–140 days after exposure, while C57Bl/6 mice present with fibrotic death after 150–200 days. We followed both lung pathologies by CT scans and all syndromes by histology.

### Chemical Synthesis of the NPSP512 Derivatives QS1 and 52A1

QS1: The synthetic approach was adapted from Laval et al. (2009) *.* 40 mmol (6.49 g) of 1-phenylpiperazine was placed in a 250 ml Erlenmeyer flask equipped with a magnetic stirrer. Subsequently, 45 ml of anhydrous tetrahydrofuran (THF), 80 mmol (8.8 ml) of 4-methylmorpholine and 50 ml of anhydrous dimethyl sulfoxide (DMSO) were added, and the solution was cooled in an ice bath for 10 min with vigorous mixing. Then, 44 mmol of appropriate sulfonyl chloride was dissolved in 25 ml of anhydrous THF, and the resultant solution added in 2.5 ml portions to the reaction mixture over a period of 10 min with mixing. The flask was covered and mixing continued overnight (∼18 h) after which the reaction mixture was transferred to a 500 ml round-bottom flask, and the THF evaporated using a rotary evaporator (bath temp 35°C). The remaining residue was diluted with water and formed precipitate was collected by filtration. Dried crude compounds were crystalized from either ethyl alcohol or acetonitrile giving corresponding compounds with overall yield of 72–98%.

52A1 (4-(4-[2(aminomethyl)phenyl]piperazine1sulfonyl)phenyl)(hydroxy)oxoammonium): In brief, 3.73 g (10 mmol) of sulfonamide was placed in a 250 ml one-neck round-bottom flask equipped with a magnetic stirrer. Subsequently, 100 ml of anhydrous benzene, 2.97 ml (10 mmol) of titanium (IV) isopropoxide (Ti(OiPr)_4_), and 8.86 ml (50 mmol) of 1,1,3,3-tetramethyldisiloxane (TMDS) was added to a glass stopper capped bottle and all mixed for 24 h (60°C). The dark brown solution was then acidified with 4N HCl in 1,4-dioxane and evaporated on rotary evaporator. Crude compound was precipitated by addition of dry diethyl ether, separated by centrifugation, and dried under the vacuum. Subsequently, solid residue was extracted with hot water (3 × 50 ml), and combined extracts lyophilized, purified crystallization and a hydrochloride salt made. The formula weight was 377.44.

### Pharmacokinetic Analyses

Pharmacokinetic data were obtained from blood plasma samples taken at indicated times after a single s.c., injection of NPSP mitigators at 5 or 10 mg/kg into C3H male mice. Plasma samples (20 µL) and an internal standard (1 µL; F512, 50 pmol) were added to methanol (79 µL, Fisher Scientific Optima^®^ LC/MS) for a total volume of 100 µL, as well as 52A1 standards (1 µL of 10, 50, 100, 250 pmol) similarly prepared. These were vortexed for 2 s and centrifuged for 5 min at 16,000 rcf to obtain supernatants that were run on a 100 × 2.1 mm C18 column (Phenomenex Kinetex) using the 1290 Infinity LC system (Agilent 6460). The mobile phase was 0.1% formic acid in Milli-Q water, and 0.1% formic acid in acetonitrile. After elution, the NPSP was characterized on a triple quadrupole LC/MS system with an electrospray ionization source (Agilent 6460). Data acquisition was made in the positive ion mode for multiple reactions monitoring (MRM). The analyte signal was normalized to the internal standard and concentrations were determined by comparison to the calibration curves for plasma using linear regression analysis. PK values were obtained using SummitPk software to calculate Cmax and T1/2.

### Statistics

Kaplan-Meier plots with log rank statistics were used to test for significant survival differences. Probit analyses were performed using SPSS v20 software and NCSS PASS 13 Power Analysis and Sample Size Software, Kaysville, Utah for power analysis. Analyses of variance were performed on all other data with a Brown-Forsythe test where homogeneity of variance assumptions were not met. Multiple comparisons procedures using Sidak were also performed. The Kruskal-Wallis non-parametric test was performed for some data distributions with less stringent assumptions. Significance was assessed at the 5% level using SPSS v20 software (IBM SPSS Statistics, Armonk, NY, United States).

## Results

### *In Vitro* Screening

**FIGURE 1:**
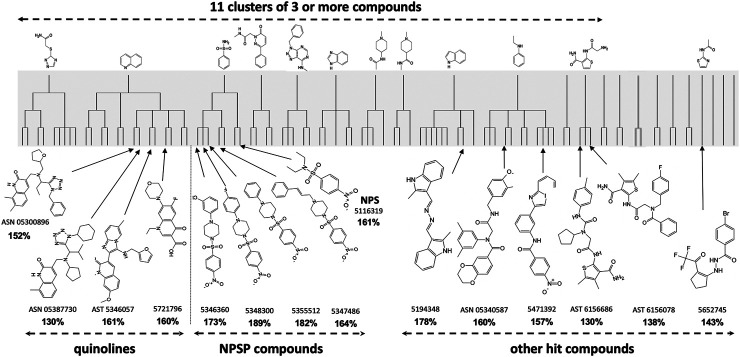
Maximal Common Substructure analysis of *in vitro* HTS “hits” (shaded) showing clusters of at least three active compounds. The 15 validated hits cherry-picked for *in vivo* testing are shown along with the extent to which they mitigated 2 Gy *in vitro* radiation cytotoxicity (in bold) compared to controls treated with 2 Gy alone (100%).

Primary HTS of the chemical libraries identified 220 potential “hits” that enhanced lymphocytic cell survival to >130% which was outside the 99% confidence limits of the control. Maximal common substructure analysis classified most of these hits to fall within 11 clusters, each with a common core pharmacophore and containing at least three active compounds (Figure 1–shaded). Secondary screening validated 23 compounds, of which 15 were cherry-picked for *in vivo* testing for mitigation of H-ARS in mice; the others being difficult to obtain. Four had a quinoline scaffold and came from the largest cluster (a total of 14 compounds at four levels of similarity). Five bore a 4-nitrophenylsulfonamide motif. All compounds had very little activity on non-irradiated cells suggesting some selectivity for radiation damage.

### *In Vivo* Screening

H-ARS was induced in C3H or C57Bl/6 male or female mice by WBI with LD_70/30_ doses estimated from institutional probit curves. Unless otherwise stated, all drugs were tested using the same schedule of five daily s. c. injections starting 1 d after LD_70/30_ doses of WBI, most at three different doses unless limited by availability. All 15 compounds shown in Figure 1 mitigated H-ARS at at least one dose regimen without observed toxicity.

### 4-(Nitrophenylsulfonyl)-4-Phenylpiperazine Compounds

We previously reported on a novel and unique class of 4-nitrophenylsulfonamide (NPS) H-ARS mitigators, four of which were 4-(nitrophenylsulfonyl)-4-phenylpiperazines (NPSP) (Micewicz et al., 2017). Lead NPS and NPSP compounds effectively mitigated H-ARS in both strains of mice and both sexes. The lead NPSP compound 5355512 [a.k.a. 512 or #5 (Bhat et al., 2019)] mitigated multiple radiation syndromes.

**FIGURE 2:**
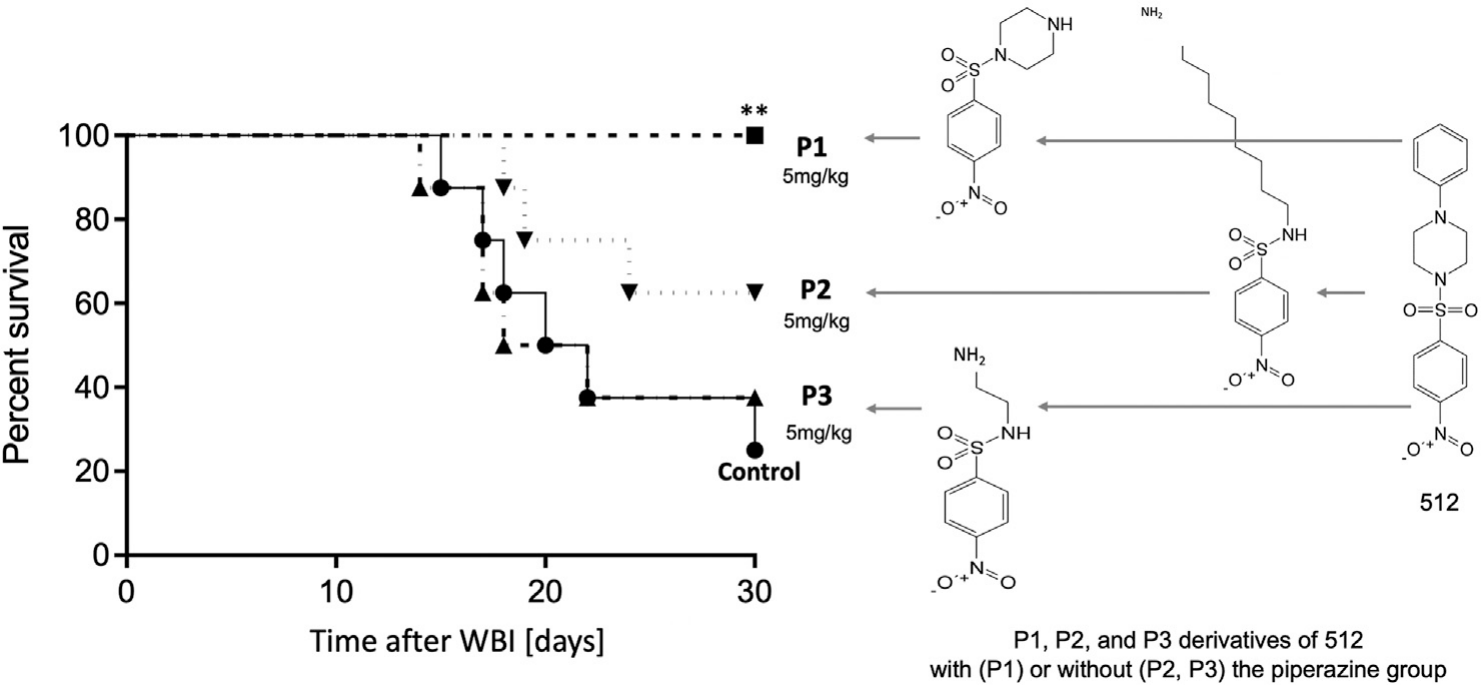
The piperazine group is essential for efficacy of 512 at 5 mg/kg, but can be modified without loss of activity, P1 being able to fully mitigate H-ARS (*p* = 0.0085) whereas P2 and P3 analogs without piperazine lost activity.

The shared nitrophenylsulphonamide moiety obviously is the active warhead for this class of drugs, but we chose a piperazine derivative as the lead compound because of its more favorable PK properties (Micewicz et al., 2017). We hypothesize that this enabled activity at 5mg/kg, which is easier to deliver to humans than the 75 mg/kg required for activity of simpler NPS compounds (Micewicz et al., 2017). In keeping with this hypothesis, analogs of NPSP512 that we synthesized without piperazine lost low dose (5 mg/kg) efficacy (Figure 2). Although the piperazine group could be modified with retained activity, not all NPSPs in our HTS dataset with >70% similarity were active mitigators and this appeared to be due to subtle chemical features associated with the piperazine group (Micewicz et al., 2017).

### Formulation, Quaternization, and Derivatization of 512

For preliminary *in vivo* studies, drugs were dissolved in DMSO and formulated in Cremophor EL to minimize inter-drug differences in solubility. However, high concentrations of Cremophor have been reported to be associated with occasional hypersensitivity reactions in patients (Gelderblom et al., 2001), and the FDA now consider it an undesirable excipient. NPSP512 needed to be formulated for further advancement. Dozens of FDA-approved excipients were tested without much success. By analogy with x-ray crystallography of N-(4-nitrophenyl)-N-phenylsulfonamide (Gomes et al., 1993), it seems that NPSPs can “stack” head to tail in alternate hydrophobic and hydrophilic layers to form large intermolecular complexes that “crash out” over time in aqueous solution. This was not a problem in our preliminary studies because solutions for injection were made fresh before use. Since we could find no excipient that could overcome this problem, we resorted to synthesis of chemically modified analogs. Guided by the extensive SAR studies, we knew that the groups attached to piperazine most likely affected solubility and chose this as the target for modification.

**FIGURE 3:**
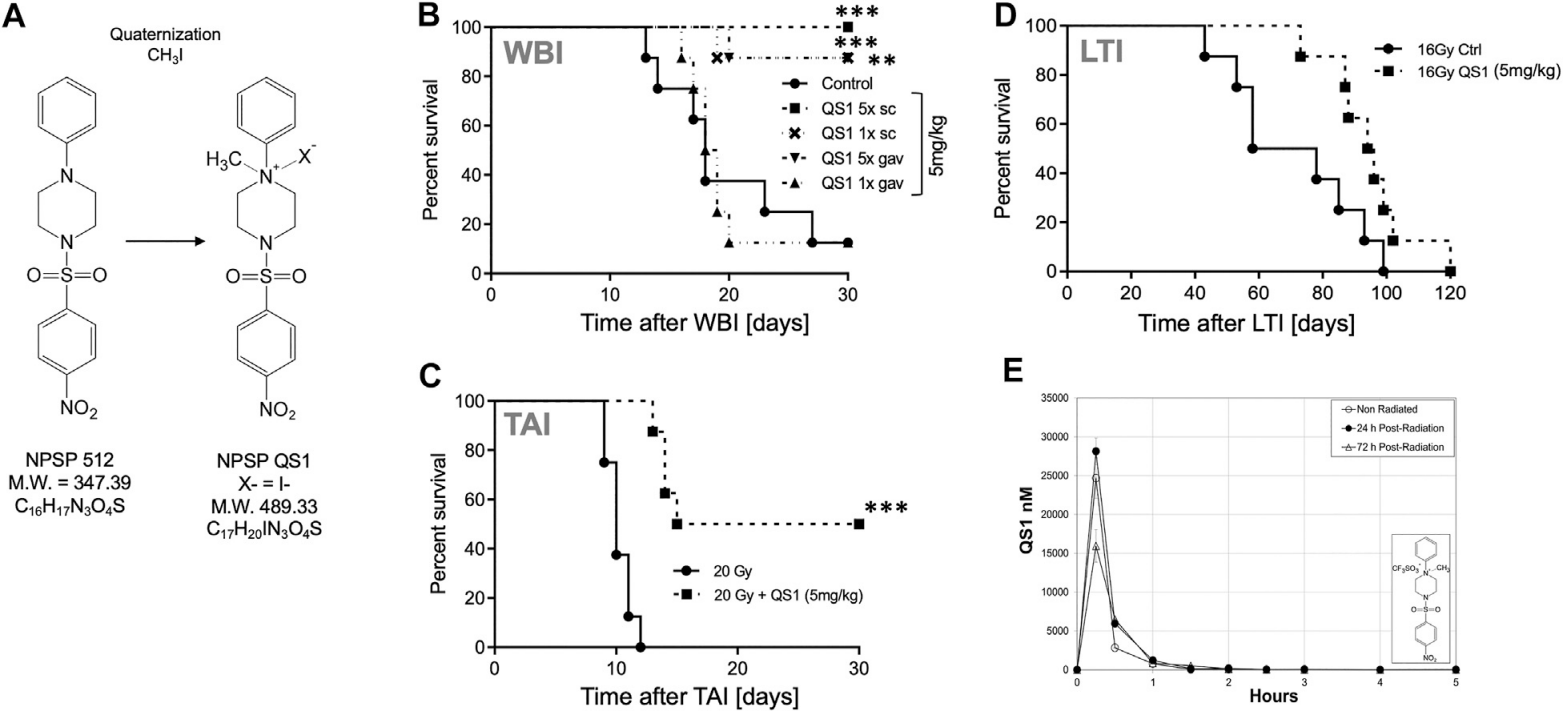
**(A)** Quaternization of NPSP512 to yield QS1. **(B)** QS1 at 5 mg/kg mitigates H-ARS lethality in C3H male mice after LD70/30 WBI (*p* < 0.0001; Cs source; 7.725 Gy) if given 5× daily by s.c., or gavage, or 1× if given s.c., (*p* = 0.0023), but not by gavage. **(C)** QS1 at 5 mg/kg, 5X daily s.c., mitigates GI-ARS after 20 Gy total abdominal irradiation in C57BL/6 male mice and **(D)** radiation pneumonitis in C3H mice after 16 Gy local thoracic irradiation. **(E)** PK parameters for 10 mg/kg QS1 injected s.c., assessed when drug is given 24 or 72 h after WBI showing a decrease in plasma concentration if given at 72 h. * = *p* < 0.05, **p < 0.01, ***p < 0.001.

NPSP512 was quaternized as shown in Figure 3A and designated QS1. QS1 was 50 times more soluble in water than NPSP512 reaching 2.4 mg/ml. Although not shown here, like the parent molecule, QS1 mitigated radiation-induced apoptosis and enhanced mobilization of CD11b^+^Ly6G^+^Ly6C^+^ myeloid cells (Micewicz et al., 2017). The optimal dose of QS1 needed for mitigating H-ARS lethality in C3H mice was determined using 1, 5, 10, or 20 mg/kg injected s. c., 5× daily starting 1 d after an LD_70/30_ WBI dose and found to be 5 mg/kg (not shown)—identical to that for the parent compound (Micewicz et al., 2017). In several different H-ARS experiments, 5 mg/kg s.c., (5x) increased survival from 30% to 90–100%, a regime that was also effective for gavage delivery (Figure 3B). A single injection of QS1 24 h after WBI was also effective if given s. c., but not by gavage. Like the parent 512, the standard 5 d schedule of 5 mg/kg s. c. mitigated GI-ARS lethality in mice receiving 20 Gy to the abdomen (Figure 3C) and radiation pneumonitis after 16 Gy to the thorax (Figure 3D). PK studies (Figure 3E) showed that the C_max_ for QS1 was almost 100-fold higher than for NPSP512 but that it had a very short half-life and could not be detected in plasma for longer than 3–4 h after injection, likely due to its metabolism. For comparison, the C_max_ for NPSP512 occurs after 2 h and drug is detectable for up to 24 h (Micewicz et al., 2017). The PK characteristics were very similar in non-irradiated and WBI mice when drug was injected 24 h after WBI, but when injected 72 h post-WBI the C_max_ was lower indicating radiation changes the plasma fluid volume over time.

**FIGURE 4:**
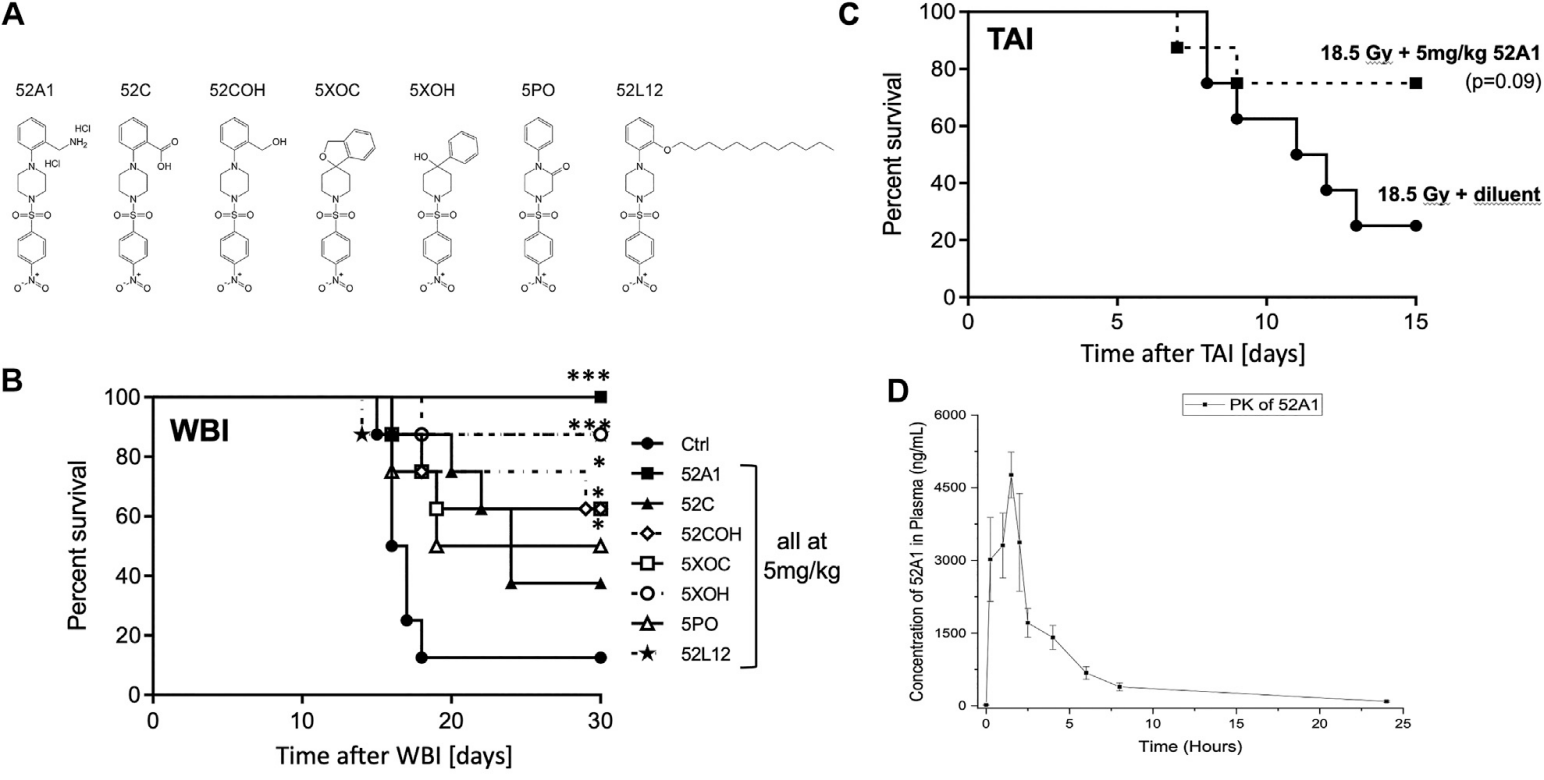
**(A)** 512 derivatives synthesized and tested in **(B)** a model of H-ARS using LD70/30 doses (X-rays) in C3H mice. Drugs were given 5× s.c., except for the lipidated 52L12, which was given by gavage in Kollopher EL. The water soluble 2-(aminomethyl)-derivative of 512, 52A1, was most effective. **(C)** 52A1 also mitigated GI-ARS in C57Bl/6 mice given 18.5 Gy WBI with 2.5% body shielding. **(D)** PK of 52A1 is similar in shape to 512 but the Cmax is almost 40 times higher. * = *p* < 0.05, **p < 0.01, ***p < 0.001.

Although QS1 solubility was improved 50 fold by quaternization, its bioavailability became suboptimal compared to the parent molecule. Over 90 derivatives of NPSP512 were therefore synthesized in attempts to optimize its pharmacology with respect to a) viable substituents for the -NO_2_ group, b) solubility in water, and c) enhanced oral bioavailability using lipidation to improve intestinal absorption (Madsen et al., 2007; Bellmann-Sickert et al., 2011). All were screened for anti-apoptotic activity *in vitro* and seven of the most promising were tested for ability to mitigate H-ARS *in vivo* (Figure 4). The water-soluble 2-(aminomethyl)-derivative of NPSP512, 52A1, emerged with greatly improved pharmacology while still being highly effective at mitigating lethality from H-ARS in C3H mice (Figure 4B). 52A1’s ability to mitigate GI-ARS in C57Bl/6 mice was also robust albeit just below reaching statistical significance (Figure 4C, *p* = 0.09) with the new FDA-preferred GI-ARS model, which is WBI with one leg shielded (2.5% bone marrow shielding) (Farese et al., 2019). Note that the MST for GI-ARS is delayed in gnotobiotic over more conventional mice (Wilson, 1963; Matsuzawa, 1965), and that 18.5 Gy is a high dose GI-ARS model that is not bone marrow-dependent, unlike lower dose models (Mason et al., 1989). The PK of 52A1 was very similar to NPSP512, but with an almost 40-fold higher C_max_ (Figure 4D). Lipidation of NPSP512 yielded a water insoluble hydrophobic compound that was suspended in Kollipher for oral delivery. The length of the fatty acid chain is an important parameter for improving bioavailability (Madsen et al., 2007). Derivatives with chains of up to 20-carbon fatty acids were synthesized and tested by the oral route (not shown). The 12-carbon molecule, 52L12 was the most effective (*p* < 0.001; Figure 4B).

### Quinoline Compounds

**FIGURE 5:**
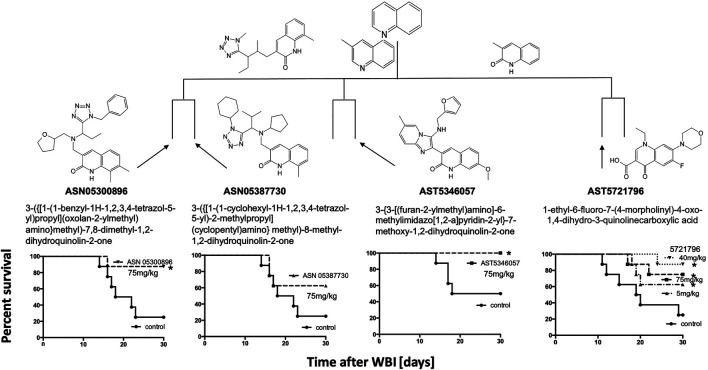
Activity of the quinoline class of radiomitigators of H-ARS in C3H male mice with drugs given s.c., at the stated doses (5× daily) starting 1 d after LD70/30 doses of radiation (Cs-137). *p < 0.05, ***p < 0.001.

The quinoline class detected by maximal common subclustering had four compounds that stood out after secondary screening. Their ability to inhibit radiation killing *in vitro* is shown in bold in Figure 1 and their ability to mitigate H-ARS is shown in Figure 5. Although drug availability and therefore dose response data was limited for three compounds, at least three of the four had significant *in vivo* activity at 75 mg/kg and lower doses seemed less effective, where tested. One of the compounds in Figure 5, 5346057 (a.k.a. 057, BCN057, or YEL002), was of interest because it was identified as active in another HTS yeast screen, performed in parallel based on the DEL assay that measures mitigation of radiation cytotoxicity and genotoxity in the diploid yeast *S. cerevisiae* strain RS112 (Hafer et al., 2010; Kim et al., 2011) indicating that a conserved pathway is involved in mitigation. 057 had a dose modification factor for H-ARS of 1.15 when given s. c. in a daily 5 day course starting 24 h after WBI (Drs. Y. Revina, R. Schiestl pers. comm.). These drugs are very hydrophobic, which may be why high doses are needed for bioavailability. We had previously formulated 057 in 30% Captisol and shown that 90 mg/kg BCN057 given daily s.c., for 8 d starting on day +1 after 14, 15, and 16 Gy total abdominal irradiation mitigated GI-ARS in C57Bl/6 mice by enhancing regeneration of Lgr5+ intestinal stem cells *in vivo* and in organoid culture (Bhanja et al., 2018). The same compound, as YEL002, also decreased ^(131)^I-induced DNA double strand breaks in thyroid cells (Hershman et al., 2011) and prevented radiation-induced carcinogenesis in mice (RS, pers. comm.). The lead quinoline therefore seems active in more than one model of radiation damage and this is likely true for the whole class given their structural similarity.

### Other HTS Compounds

**FIGURE 6:**
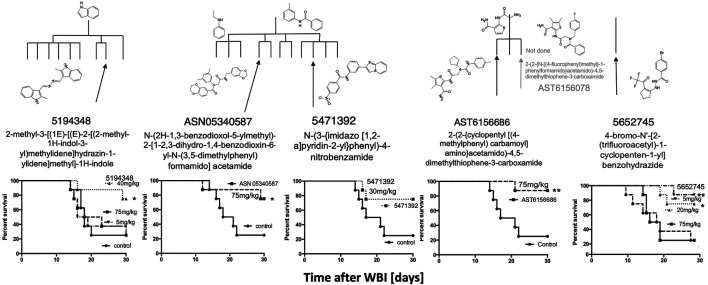
Five other HTS *in vitro* “hits” mitigated H-ARS in C3H mice given once daily for 5 d starting 24 h after LD70/30 doses of WBI (Cs). * *p* < 0.05, **p < 0.01, ***p < 0.001.

Since the two major groups of the most promising compounds in the HTS showed mitigator activity *in vivo*, “hit” compounds from the other groups (*n* = 6) with diverse structures shown in Figure 1 were also synthesized and tested for their ability to mitigate H-ARS *in vivo* after LD70/30 radiation doses (Figure 6). Although studies were limited by compound availability to five of these, all had some mitigating ability, and all but one reached statistical significance.

**FIGURE 7:**
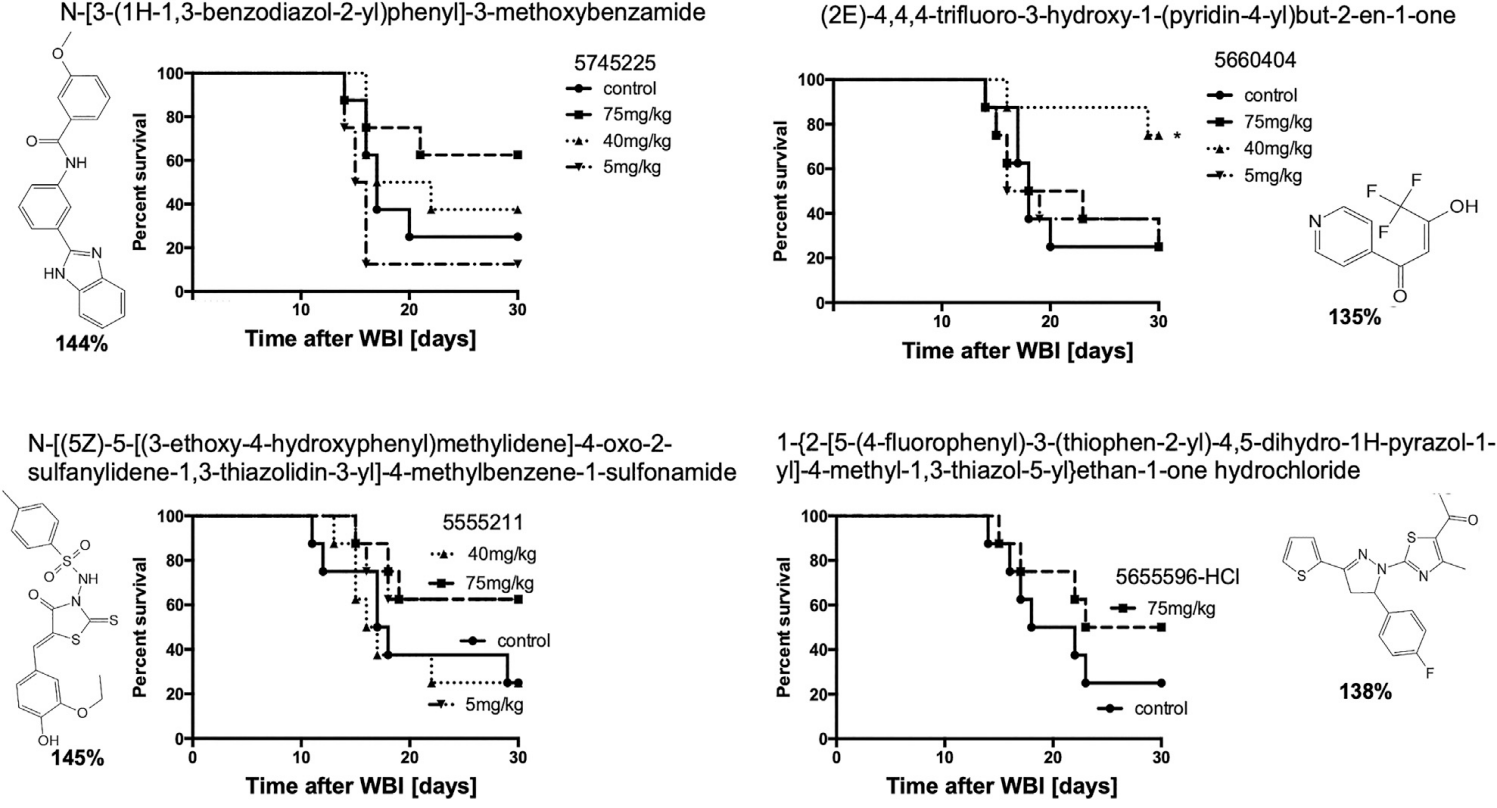
Other, lesser HTS “hits” tested in C3H mice with 5X daily drug doses, as stated, starting 24 h after WBI at LD70/30 doses. *p < 0.05.

To determine if compounds that were suboptimal during the initial *in vitro* screen (and below the potency of the 15 prime candidates), could still be active against H-ARS, we picked and tested four based on the fact that these four had increased *in vitro* cell survival to 135–145% and fell within the 122 compounds initially identified by HTS. The *in vitro* data are shown in bold in Figure 7. There was some efficacy, but only one was statistically significant and only at one concentration (Figure 7), which validated our selection process.

### Other Classes of Mitigators

#### Purine Nucleosides

We previously reported that the nucleoside adenosine, its derivative deoxyadenosine, and its analog vidarabine, which are 97% similar, were hits in our earlier bioactive HTS screen and showed some ability to mitigate H-ARS in mice (Kim et al., 2011). Nucleotides have a very short half-life *in vivo* but agonists and antagonists specific for one or more of the four known adenosine receptors, A1, A2a, A2b, and A3, have been generated with effects ranging from pro-to-anti-inflammatory and aiding-to-limiting bone marrow recovery. We have investigated whether they might have better properties as H-ARS mitigators. . The A1 and A2 receptor agonist 5′-(N-ethylcarboxamido)adenosine (NECA) at 0.1 mg/kg s.c., (5x) in our standard H-ARS model increased survival of C3H male mice after 8 Gy WBI from 0 to 50%. The A2 agonist CGS21680 at 2 mg/kg s. c. (5x) increased survival of C57Bl/6 mice after 9 Gy WBI from 20 to 80% and of C3H mice after 8 Gy WBI from 0 to 25% (not shown). However, the A3 agonist 2-Cl-IB-MECA, the A2 agonist CV1808 at 1 mg/kg, and IB-MECA at 1 mg/kg, were ineffective. Although these studies were limited, there was little evidence that these compounds were sufficiently superior to warrant further development as sole mitigators.

#### Natural Disaccharides

**FIGURE 8:**
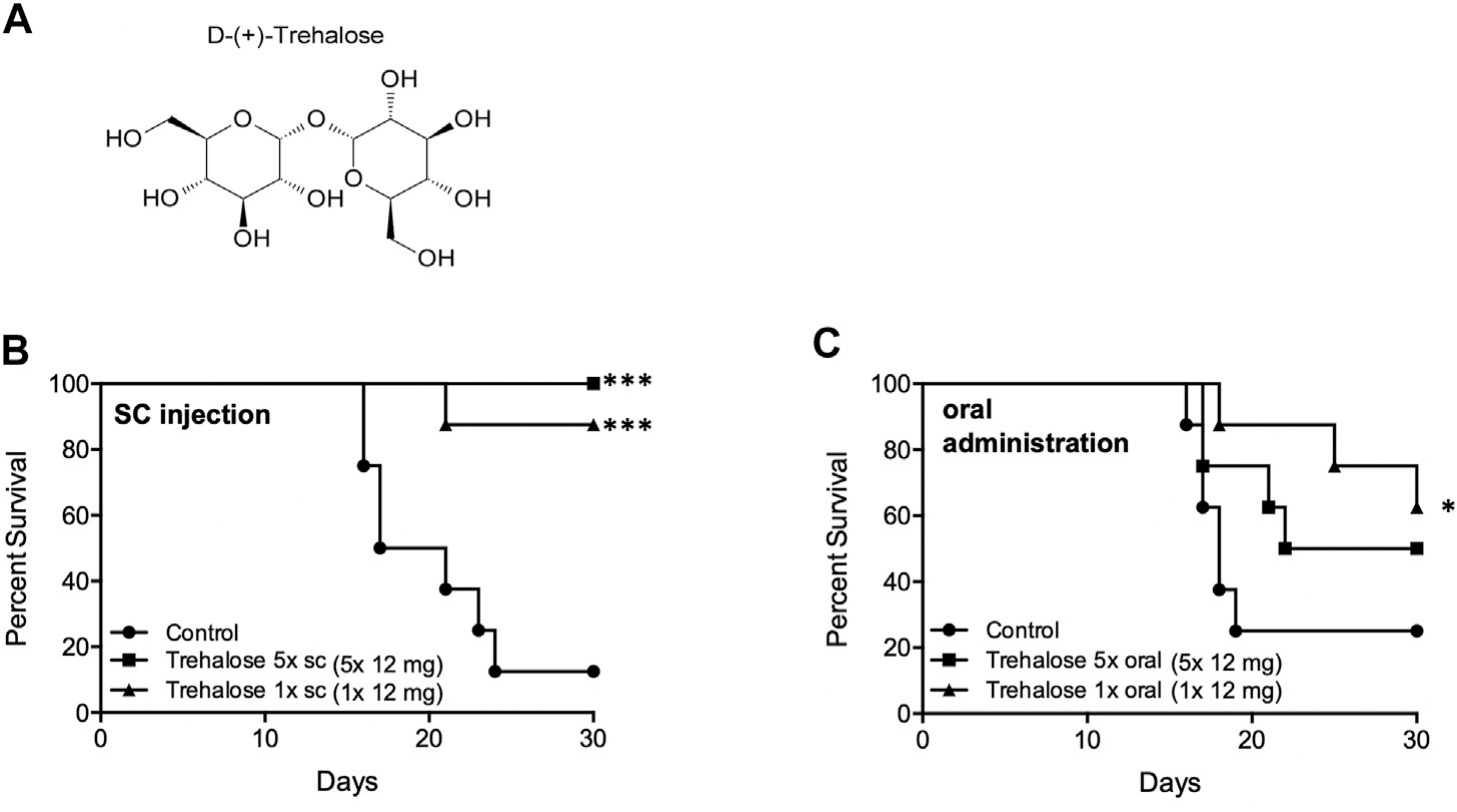
Trehalose **(A)** and mitigation of radiation damage by **(B)** Trehalose (s.c., LD70/30), **(C)** Trehalose (oral, LD70/30). Each dose was 12 mg/animal in water. *p < 0.05, ***p < 0.001.

Natural disaccharides, in particular D-(+)-trehalose, have been shown to protect DNA, cells, and organisms against UVB (Cejková et al., 2010; Cejková et al., 2012; Emanuele et al., 2014) and ionizing (Ledney et al., 1992; Yoshinaga et al., 1997; Webb and DiRuggiero, 2012; Liu et al., 2017; Paithankar et al., 2018) radiation damage, although few studies have been performed in mammalian systems. Trehalose is a multifunctional molecule that can act as an antioxidant (Elbein et al., 2003; Cejková et al., 2010) and anti-inflammatory agent (Ledney et al., 1992; Cejková et al., 2011), reducing stress in multiple models. We had included it in excipient testing for NPSP512, only to find that alone it mitigated H-ARS lethality. Animal survival was increased by both single and five daily doses of 400 mg/kg trehalose/mouse administered 24 h after WBI by the s. c. route, and to a lesser extent orally (Figure 8). Comparison with several other saccharides (sucrose, raffinose, maltose, lactose) and carnosine, suggested that trehalose is superior at mitigation. Although the administered dose was high (∼12 mg/mouse), its solubility (68.9 g/100 g in H_2_O) (Higashiyama, 2002), ready availability, high thermostability, and established lack of toxicity—it being a component of various pharmaceutical formulations and a frequent food additive—makes it a particularly intriguing candidate for clinical development.

## Discussion

We have used maximal common substructure analysis to classify 220 drugs capable of increasing lymphocytic survival *in vitro* 24 h after exposure to 2 Gy ionizing radiation. Remarkably, almost all of these could be attributed to 11 clusters of three or more compounds. Further screening to select for the most active compounds resulted in 15 drugs to test *in vivo*, and in spite of their diverse structures, almost all of these mitigated against H-ARS *in vivo*. Five had a 4-nitrophenylsulfonamide (NPS) group in common that is likely the active moiety. Here we showed that a piperazine moiety in 4-nitrophenylsulfonamidepiperazines (NPSP) improved the pharmacokinetics making the drug active at lower concentrations. Our lead NPSP, and all active agents in this class, act broadly by mitigating multiple acute and late radiation syndromes (Micewicz et al., 2017; Bhat et al., 2020; Duhachek-Muggy et al., 2020) at doses convenient for human administration. This is important because all tissues suffer radiation damage after WBI.

Unfortunately, these studies were performed with the drug formulated in Cremophor, which the FDA considers an undesirable excipient. The sulfonamide pharmacophore has been used as a drug since the 1930s and is a current component of well over 100 approved drugs with anti-bacterial, anti-cancer, anti-carbonic anhydrase, anti-diabetic, anti-protease, anti-inflammatory, and many other activities (Supuran, 2017); to which radiation mitigation can now be added. Given their popularity, the lack of literature on their solubility and lipophilicity, which are key parameters for drug development and utilization, is puzzling (Perlovich et al., 2014). Formulation attempts to find suitable excipients for NPSPs unexpectedly failed and can be explained by the tendency for these structures to “stack” and crash out of solution over time, but we were able to overcome these limitations by synthesizing alternative structures QS1 and 52A1 that retained their mitigation properties while having markedly improved water solubility and bioavailability.

Four of the 15 chemicals selected for further study were quinolines, which is another versatile scaffold for drug development forming a broad spectrum of drugs with anti-microbial, anti-inflammatory, anti-diabetic, anti-cancer, anti-malarial, anti-kinase, and other activities (Hussaini, 2016). Three of the four quinolines are shown here to have significant activity as H-ARS mitigators. These compounds are even more hydrophobic than NPSP512 but the lead compound 057 could be formulated in Captisol™ and has been shown to also mitigate H-ARS (Bhanja et al., 2018) and other radiation-induced conditions (R. Schiestl, pers. comm.). Another 4 of the 15 selected compounds, which were of diverse structures, were also active against H-ARS, as was Trehalose, which we tested as a possible excipient rather than a mitigator.

This study raises the question as to why a 24 h HTS screen using apoptosis by lymphocytic cells after 2 Gy irradiation should yield multiple mitigators of H-ARS that can be classified and grouped by common chemical moieties, and why at least some of these compounds can, additionally to being effective against hematopoietic death can also mitigate damage to epithelial structures in multiple tissues, where the critical cellular target for lethality is quite different. For H-ARS, in most species, damage to the myeloerythroid-restricted common progenitor cell compartment, and not the lymphocyte, is most likely to result in H-ARS, and transfer of these cells is sufficient to prevent H-ARS, bridging the developmental gap until more pluripotential stem cells develop (Na Nakorn et al., 2002). The FDA-approved mitigator, G-CSF is thought to act against H-ARS by acting as a survival and proliferative signal for this population (Williams et al., 1990), although it can also decrease reactive oxygen species (ROS) and prevent apoptosis through activating the anti-oxidant, anti-inflammatory Nrf2 pathway (Yamaguchi et al., 2020). For GI-ARS, Lgr5^+^ epithelial cells in the base of the crypt appear to be the weak link (Hua et al., 2012). In this case, a quiescent, Bmi1+ “reserve” stem cell population (Tian et al., 2011; Yan et al., 2012) may repopulate empty stem cell niches after injury, reprograming themselves as Lgr5^+^ cells (Tetteh et al., 2015; Wabik and Jones, 2015; Beumer and Clevers, 2016; Jones and Dempsey, 2016; Gehart and Clevers, 2019).

Our HTS discovery of multiple classes of ARS mitigators using radiation-induced cell death of lymphocytic cells as targets might implicate a role for apoptosis in ARS. However, mitigators are given 24 h after IR *in vivo*, when most rapid p53-dependent apoptosis is over. A second wave of radiation-induced, p53-independent apoptosis does occur as do later waves of ROS production and inflammation and those are the much more likely mitigator targets (Schaue and McBride, 2010; Schaue et al., 2015). Pro-inflammatory cytokines are generated through radiation-activated NF-κB and MAPK pathways, the classic DSB-initiated DNA damage response, and nucleic acid sensing pathways downstream of micronuclei and cytoplasmic nucleic acids that are increased after IR (Maelfait et al., 2020). From an evolutionary perspective, inflammatory programs enacted in immune and non-immune cells aim to remove potential pathogens, but after WBI they come at a risk of exacerbating radiation damage.

**FIGURE 9:**
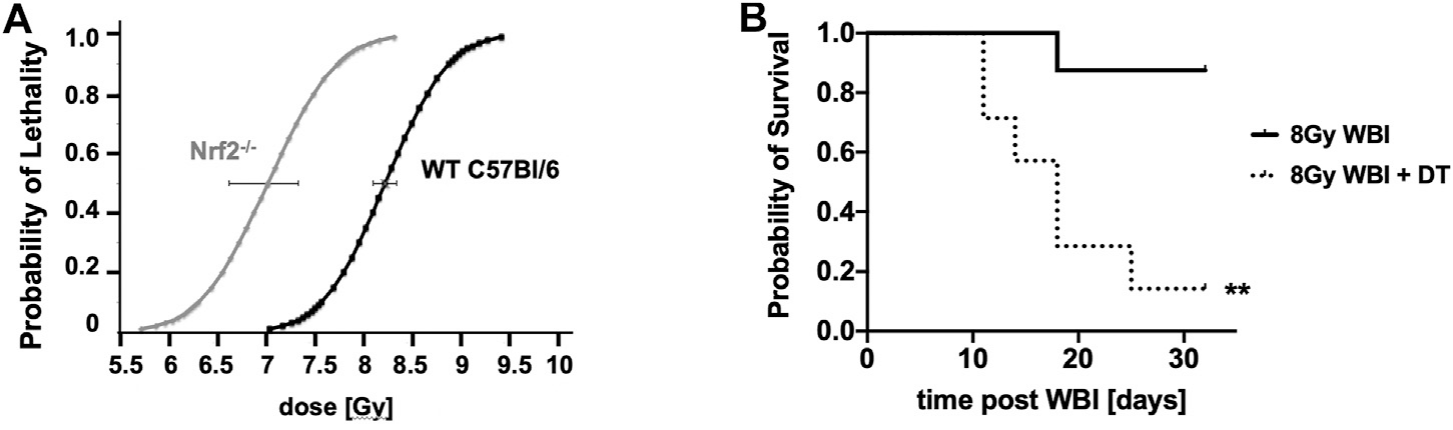
The impact of Nrf2 depletion **(A)** and Treg depletion in Diphtheria toxin (DT)-treated Foxp3DTR/EGFP transgenic mice **(B)** (**p = 0.0037) on responses to WBI compared to aged-matched controls. **p < 0.01.

This simple view must be tempered by the fact that pro-oxidant, pro-inflammatory milieus provoke an anti-inflammatory antagonistic response, often involving the anti-oxidant Nrf2 pathway. Mechanistically it is still not entirely known how tissues transition to an anti-inflammatory state that can support cell proliferation and restore tissue function, but it is clear that healing is compromised by RT and that radiation disease evolves over time with cyclical changes in inflammatory/anti-inflammatory pathways and in redox and metabolic status (Schaue et al., 2015). The contribution of Nrf2 can be judged by the fact that mice with the Nrf2 pathway deleted are about 15% more radiosensitive to WBI than wild type C57Bl/6 mice (Figure 9), which is in keeping with the finding that NRF2-mediated Notch signaling improves hematopoietic stem/progenitor function after IR (Kim et al., 2014). Similarly, depletion of anti-inflammatory Tregs increases the pro-inflammatory response to IR (Schaue et al., 2012) and radiosensitizes C57Bl/6 mice to WBI (Figure 9). In fact, when studied, all of the mitigators mentioned in this paper, and others like G-CSF (Yamaguchi et al., 2020), quench radiation-induced inflammation and ROS production (Kim et al., 2009; Micewicz et al., 2017; Bhat et al., 2020) and it is reasonable to speculate that this is a requirement for effective mitigation. However, this is not the complete story, as can be seen from the other properties of the mitigator classes we discovered. Tissue recovery after injury engages many complex, interactive, highly conserved pathways within an entire responsive biological network which offers many possible points of intervention.

Both lead compounds from these two major classes of mitigators, the NPSPs and quinolones, where found to stimulate proliferation in stem cell organoid cultures of intestine, brain, lung and bone marrow (Micewicz et al., 2017; Bhanja et al., 2018; Bhat et al., 2019; Bhat et al., 2020; Duhachek-Muggy et al., 2020). This includes Lgr5+ stem cells present in steady state and during post-injury tissue regeneration in the intestinal crypts, and other tissues. The balance between self-renewal and differentiation in these stem/progenitor cells is tightly controlled by a complex interplay of evolutionary conserved signaling pathways, including Wnt, Notch, bone morphogenetic protein, and Hedgehog (Clevers, 2019). Smoothened, a key component of the sonic hedgehog (SHH) signaling pathway is activated in the gut by NPSP 512 leading to nuclear translocation of the GLI1 transcriptional activator to mitigate GI-ARS (Duhachek-Muggy et al., 2020). Further, the quinoline 057 activates canonical Wnt-β-catenin signaling to mitigate GI-ARS (Bhanja et al., 2018) and extracellular vesicular delivery of Wnt rescues intestinal stem cells from radiation toxicity (Saha et al., 2016). It is reasonable to suggest that these agents may work through the same integrated signaling network. The SHH and Wnt signaling pathways are closely linked and, with Bmi-1, are involved in regulating embryonic and post-natal development, and stem cell proliferation (Ouspenskaia et al., 2016) to affect tissue repair and regeneration (Singh et al., 2018; Cao et al., 2020). We speculate that these developmental signaling pathways are reactivated in many tissues to promote tissue recovery and that mitigators can promote this process. Our colleagues (Himburg et al., 2017) have shown that, while IR activated canonical Wnt signaling, the Wnt antagonist Dickkopf-1 (Dkk1) mitigated H-ARS and promoted recovery of both long-term repopulating hematopoietic stem and progenitor cells, both directly and through niche interactions perhaps illustrating the complexity inherent in these pathways.

Others have reported that inhibition of the Wnt activator GSK-3β by SB21676324 mitigated H-ARS in C57Bl/6 male, but not female C57Bl/6 or C3H mice, indicating sex and strain differences (Daniel et al., 2020). The only sex difference we found was that NPSP512 stimulated neural progenitor populations in female, but not male mice (Bhat et al., 2020). Investigation of these pathways with a view to reconciling divergent results will need to take into account differences between tissues and sexes, comparison of varying radiation doses and administration schedules, and the degree of canonical activation of the Wnt pathway, which substantially impacts stem cell proliferation and differentiation (Luis et al., 2011). It is worth noting that Wnt signaling also provides crucial proliferative and survival signals to immature T cells (Ma et al., 2012), and our use of such cells for mitigator screening may have inadvertently biased our HTS towards agents that increase cell survival through activating these signaling pathways.

We suggest that the mitigators described here have, in addition to anti-inflammatory properties, the ability to enhance stem cell signaling through elements of the Hedgehog/Patch/Smoothened/Gli, BMP/TGF-β, FGF, WNT and Notch networks that govern embryogenesis, homeostasis and repair of adult tissues (Katoh and Katoh, 2006; Amankulor et al., 2009; Pitter et al., 2014; Caradu et al., 2018; Guo et al., 2018; Moparthi and Koch, 2019; Zhou et al., 2019). As far as mitigation of radiation damage is concerned, the enormous complexity inherent in these networks and the importance of context indicate a need for much further consideration as to mechanism. However, it should be pointed out that there is a close relationship between pathways controlling tissue repair and regeneration and inflammation, which may account for why these two aspects appear to be linked as common properties shared by classes of mitigators (Omenetti and Diehl, 2008; Amankulor et al., 2009; de la Roche et al., 2013; Braune et al., 2017; Shen et al., 2017; Caradu et al., 2018; Guo et al., 2018; Razumilava et al., 2018).

## Data Availability

The original contributions presented in the study are included in the article/Supplementary Material, further inquiries can be directed to the corresponding author.
